# A paradigm shift in psoriasis treatment: deucravacitinib’s significance

**DOI:** 10.1097/MS9.0000000000001484

**Published:** 2023-11-07

**Authors:** Galib M. Abrar Ishtiaque, Fatema A. Supti, Ruhul Amin, Talha B. Emran

**Affiliations:** a Department of Pharmacy, Faculty of Allied Health Sciences, Daffodil International University, Dhaka, Bangladesh; b Department of Pharmacy, BGC Trust University Bangladesh, Chittagong, Bangladesh; c Faculty of Pharmaceutical Science, Assam Down Town University, Panikhaiti, Guwahati, Assam, India

*Dear Editor,*

The term ‘small molecule’ refers to organic compounds with a low molecular weight. The pharmaceutical industry relies heavily on them and has been doing so for a long time. Small molecules are useful therapeutic drugs for numerous reasons, including their orally bioavailable nature and ability to cross cell membranes. Furthermore, features like high affinity and selectivity can be engineered into small-molecule drugs^1^. One such small molecule, deucravacitinib (Sotyktu), an oral tyrosine kinase 2 (TYK2) inhibitor, is extremely specific and the first of its class. This is achieved via an allosteric mechanism that maintains an antagonistic connection across the catalytic and regulatory domains of TYK2’s catalytically inactive pseudokinase regulatory domain. Janus kinases, such as Janus kinase (JAK) 1 (JAK1), JAK2, and JAK3, as well as TYK2, are all critical components of the cytokine signaling cascade^2^. Since the interleukin (IL)-23 and IL-17 pathway has been recognized as the key pathogenic mechanism of psoriasis, TYK2 is being examined as a potential target for therapy in the management of this illness. TYK2 is essential for signal transduction to occur when IL-12, IL-23, and type I interferons are released into the body, starting a cascade of events that eventually culminates in an adaptive or innate immune response^3^. Deucravacitinib is an investigational medicine developed by Bristol Myers Squibb for the medical management of lupus, inflammatory bowel disease, psoriatic arthritis (PsA), and psoriasis. It has been authorized for use by persons in the United States (U.S.) who have moderate-to-severe plaque psoriasis since its original clearance on 9 September 2022. On the 26th of September 2022, it was granted the green light from the Pharmaceuticals and Medical Devices Agency (PMDA), Japan, making it possible to use it for the management of plaque psoriasis as well as generalized pustular and erythrodermic psoriasis^4^. The medicine termed deucravacitinib, an allosteric TYK2 inhibitor, is a pseudokinase (JAK2) inhibitor that blocks enzyme activity by attaching to the protein’s regulatory domain rather than its catalytic one. When TYK2 is compared to other tyrosine kinase enzymes, it has high selectivity because of its interaction (Fig. 1).

**Figure 1:**
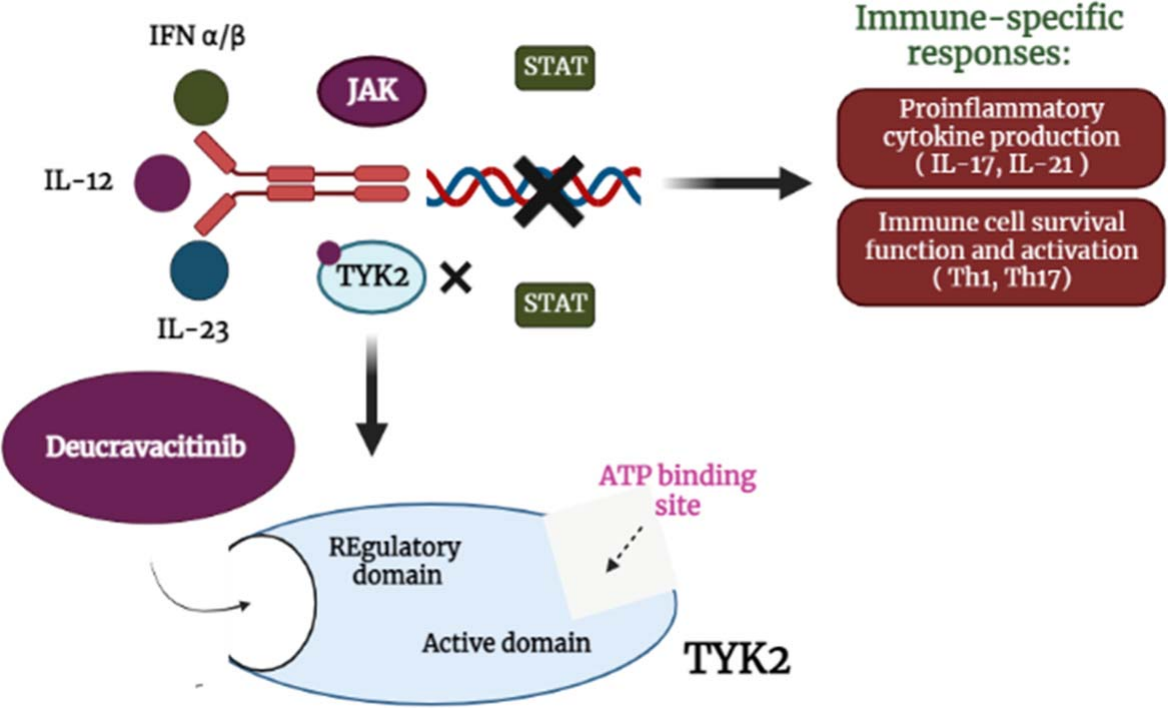
Deucravacitinib’s mode of action. ATP, adenosine triphosphate; IFN, interferon; IL, interleukin; JAK, Janus kinase; TYK, tyrosine kinase; Th, T helper; STAT, signal transducer and activator of transcription.

In comparison to other anti-JAK1/2/3 medicines, deucravacitinib is 100–2000 times more specific against TYK2 and has limited to not have any impact on JAK1/2/3. Deucravacitinib’s capacity to bind to TYK2 and form binding antagonistic confirmation between the regulatory and catalytic sections of TYK2 is what permits it to reduce TYK2 activity^5^. The IL-23, Th17 pathway, IL-12 signaling, type 1 interferon pathway, and keratinocyte activity are all negatively affected by the downregulation of TYK2. These Th17 cells produce the IL-17 and IL-22 that led to organ damage in a systemic form of lupus nephritis and erythematosus, as well as osteoclast differentiation and activation in PsA. Moreover, it is responsible for mucosal and barrier damage in inflammatory bowel disorder and keratinocyte activation and proliferation in psoriasis. TYK2 is a critical regulator of IL-23 signaling^6^. It is more specific for TYK2 when compared to other anti-JAK1/2/3 drugs. Deucravacitinib has little or no impact on any therapeutic use of JAK1–3 suppression in the circulatory system. As a result, it does not cause the negative side effects of these drugs, such as a decrease in hemoglobin levels and changes to homeostasis of platelets. Additionally, deucravacitinib does not affect the ‘IL-6 inhibition signatures’ that have been seen in response to known inhibitors of the IL-6 pathway, such as JAK1 inhibitors, which can lower the level of neutrophils and raise triglyceride levels^7^.

Deucravacitinib showed a dependent dose rise in concentration (mean *t*_1/2_e=8–13 h), a median *T*_max_ of 2–3 hours, an oral absolute bioavailability of 99%, and a rapid as well as almost complete oral absorption. *C*_max_ and area under the curve (AUC) were proportionally increased in healthy subjects after oral dosing with deucravacitinib at dosages ranging from 3 to 36 mg. The steady-state volume for deucravacitinib delivery at a dose of 10 mg/ml is 140 l. It displayed a blood plasma concentration ratio of 1.26 and was bound to proteins at a rate of 82.9–100.0%. The cytochrome P450 (CYP) 1A2 enzyme catalyzes the breakdown of deucravacitinib, which results in the formation of BMT-153261. The cytochrome P450 (CYP-450) enzymes that degrade deucravacitinib include carboxylesterase 2, uridine glucuronyl transferase 1A9, uridine cyclase, and CYP2B6. Through hepatic metabolism, rapid renal as well as fecal elimination, and other mechanisms for clearance, the body eliminates 39% of the original molecule and 59% of the metabolites. Radiation from a single oral dose of radiolabeled deucravacitinib was excreted in the urine at a rate of about 13% and in the feces at a rate of about 26%^8^.

At the outset, deucravacitinib was found to be efficacious and generally easily tolerated in a single clinical study involving 108 healthy people (83 active and 25 placebo). No significant adverse events were noticed, and both the active and placebo groups experienced nonserious adverse events at about the same frequency^9^.

A total of 267 individuals who had a medium-to-serious condition known as psoriasis were included in the trial’s second phase and were assigned at random to get either the active medication or a placebo through oral administration one time every other day, a single time daily, two times per day, a total of six times a day, or a total of twelve times throughout the day. Upper respiratory infections, headaches, nasopharyngitis, diarrhea, and nausea were common side effects^10^.

This medication was found to be highly efficacious as well as safe than a medication known as apremilast and placebo in two large, recent third-phase trials; POETYK PSO-1 and POETYK PSO-2 that included adults with plaque-like conditions called psoriasis. One-third or more of the 1686 individuals suffering from medium-to-rigorous conditions known as psoriasis who participated in these clinical studies had previously received biologic therapy (666 in PSO-1 and 1020 in PSO-2). Participants studied were randomly assigned to receive deucravacitinib (roughly 6 mg once every single day), apremilast (30 mg twice a day), or a drug called placebo (in a 2:1:1 ratio)^11^. The most commonly documented adverse effects of deucravacitinib treatment were episodes of nasopharyngitis and other infections of the upper respiratory system. More persons in the apremilast category reported these side effects compared to those in the deucravacitinib or placebo groups, but everyone experienced at least one negative impact^12^. Additionally, up to 30% of psoriasis patients might develop PsA, a chronic condition characterized by inflamed joints^13^. PsA patients seem to tolerate their therapies well and report having few or no adverse effects. In light of phase III program’s findings, studies investigating long-term response, and side-by-side comparisons with other targeted medications, deucravacitinib still needs to be evaluated for any use in the treatment of PsA^14^. Furthermore, deucravacitinib has proven to be highly efficient in the treatment of scalp psoriasis. With the help of deucravacitinib, the condition referred to as ‘scalp edema’ can be alleviated. Over the course of 16 weeks, patients with severe scalp psoriasis saw a 75–80% decrease in their Psoriasis Area and Severity Index (PASI) ratings, which justifies that deucravacitinib is an excellent systemic first-line treatment for psoriasis of the scalp.

Deucravacitinib’s safety for treating intermediate to severe psoriasis with plaques has been established. Scientific studies showed that it was more effective as well as efficient than the control group and apremilast. Patients are advised to assess the advantages and disadvantages before starting treatment because deucravacitinib’s box insert mentions that it may increase the risk of infections. This is particularly important for those who are prone to disease or frequently become sick. No one should use the drug if they have a severe or active infection^15^. Cancer patients should weigh the advantages and disadvantages of each therapy option prior to starting any course of action. The level of the body’s triglycerides, creatine phosphokinase, and liver enzymes should be monitored throughout treatment because some drugs may induce an increase in these values. People with renal impairment do not require dosage changes despite the fact that someone with severe hepatic failure should not take medication^16^. In order to establish the therapeutic efficacy and effectiveness of the compound deucravacitinib over extended time periods, additional long-time research and practical expertise are needed. Improvements in efficacy, safety, and patient convenience are only some of the many reasons why oral biologic medications are preferred over injectable biologic therapy. Patients may prefer pills since they are easier to take on the go, do not need to be stored in a specific temperature range, and reduce or eliminate the risk of immunogenicity and injection site responses. These results provide evidence in favor of adding deucravacitinib to the roster of possible therapies for an intermediate-to-rigorous condition known as psoriasis. Nonetheless, additional research into the effectiveness of deucravacitinib in treating psoriasis is unquestionably needed to validate these findings and determine its specific function in this treatment^17^.

## Ethical approval

Ethics approval was not required for this editorial.

## Consent

Consent was not required for this editorial.

## Sources of funding

Not applicable.

## Author contribution

G.M.A.I.: conceptualization, data curation, writing – original draft preparation, and writing – reviewing and editing; F.A.S. and R.A.: data curation, writing – original draft preparation, and writing – reviewing and editing; T.B.E.: writing – reviewing and editing, visualization, and supervision.

## Conflicts of interest disclosure

All authors report no conflicts of interest relevant to this article.

## Research registration unique identifying number (UIN)

1. Name of the registry: Not applicable.
2. Unique Identifying number or registration ID: Not applicable.
3. Hyperlink to your specific registration (must be publicly accessible and will be checked): Not applicable.

## Guarantor

Talha Bin Emran, PhD, Associate Professor, Department of Pharmacy, BGC Trust University Bangladesh, Chittagong 4381, Bangladesh. Tel: +88 030 3356193; fax: +88 031 2550224; mobile: +88 01819 942214; https://orcid.org/0000-0003-3188-2272; e-mail: talhabmb@bgctub.ac.bd.

## Data availability statement

Not applicable for this editorial.

## Provenance and peer review

Not commissioned, internally peer-reviewed.
